# Senotherapeutics for metabolic disease and diabetic complications

**DOI:** 10.1111/joim.70039

**Published:** 2025-11-05

**Authors:** Allyson K. Palmer, Rosa Spinelli, Larissa G. Langhi Prata, Selim Chaib, Masayoshi Suda, Tamar Tchkonia, Ulf Smith, James L. Kirkland

**Affiliations:** 1 Division of Hospital Medicine, Department of Medicine, Mayo Clinic, Rochester, Minnesota, USA; 2 Division of Medicine, Sahlgrenska Academy, University of Gothenburg, Gothenburg, Sweden; 3 Department of Translational Medical Sciences, Federico II University of Naples, Naples, Italy; 4 Center for Advanced Gerotherapeutics, Department of Medicine, Cedars-Sinai Medical Center, Los Angeles, California, USA

**Keywords:** antidiabetic agents, cellular senescence, insulin, metabolic diseases, senosensitizers, senotherapeutics

## Abstract

Metabolic diseases, including obesity, Type 2 diabetes (T2D), and metabolic syndrome, are increasingly prevalent worldwide, driven by sedentary lifestyles, aging populations, and complex genetic and environmental factors. Traditionally understood as disorders of glucose and lipid metabolism, a growing body of evidence now implicates cellular senescence as a central, age-related contributor to metabolic dysfunction. Senescent cells (SCs) accumulate in key metabolic tissues where they disrupt tissue function through the senescence-associated secretory phenotype (SASP), a pro-inflammatory and fibrogenic secretome. SASP factors exacerbate insulin resistance, chronic inflammation, and tissue remodeling, advancing the progression and complications of metabolic diseases. These insights have catalyzed the development of senotherapeutics, a class of interventions that includes senolytics (to eliminate SCs), senomorphics (to suppress SASP), and senosensitizers (to render resistant SCs more vulnerable to clearance). Although preclinical studies show promise, translation into clinical practice faces significant challenges, including identifying reliable biomarkers, understanding SC heterogeneity, and optimizing treatment timing and safety. As research advances, senotherapeutics may offer a transformative approach not only to managing metabolic diseases but also to mitigating associated comorbidities. The recognition that antidiabetic agents already in clinical use can modulate key features of senescence highlights a unique translational opportunity, suggesting that prevention of age-related metabolic disorders may be achievable with therapies already available in routine clinical practice. Medicine is poised to enter a new era in which targeting cellular senescence could fundamentally reshape the prevention and treatment of age-related metabolic disorders, offering the potential for improved healthspan and reduced disease burden across the lifespan.

## Introduction

Metabolic diseases—including obesity, Type 2 diabetes (T2D), and metabolic syndrome—represent a growing global health burden, driven by lifestyle factors, aging populations, and complex genetic and environmental interactions. Although these conditions are traditionally viewed through the lens of disrupted glucose and lipid metabolism, emerging evidence points to the accumulation of senescent cells (SCs) as a critical underlying mechanism that links aging processes to the pathogenesis of metabolic dysfunction. SCs can accumulate in metabolic tissues such as adipose tissue (AT), liver, pancreas, muscle, and vascular endothelium, where they impair tissue homeostasis through their pro-inflammatory secretome—the senescence-associated secretory phenotype (SASP). SASP factors produced by some SCs promote insulin resistance, chronic inflammation, fibrosis, cancers, and organ dysfunction, suggesting that SC accumulation is not merely a marker of metabolic diseases, but a driver of their progression and complications. As a result, targeting SCs with senotherapeutics—including senolytics (agents that selectively eliminate SCs), senomorphics (agents that suppress the harmful effects of the SASP), and senosensitizers/chemosensitizers (agents that convert essentially silent, senolytic/chemotherapy-resistant SCs into cells that can be removed by senolytics, chemotherapy, and the immune system)—has emerged as a promising therapeutic approach. This review explores the growing body of research connecting SCs to metabolic disease and highlights the translational potential of senotherapeutics to reshape how we understand and treat these age-related conditions.

### Mechanisms of senescent cell formation and propagation

**Fig. 1:**
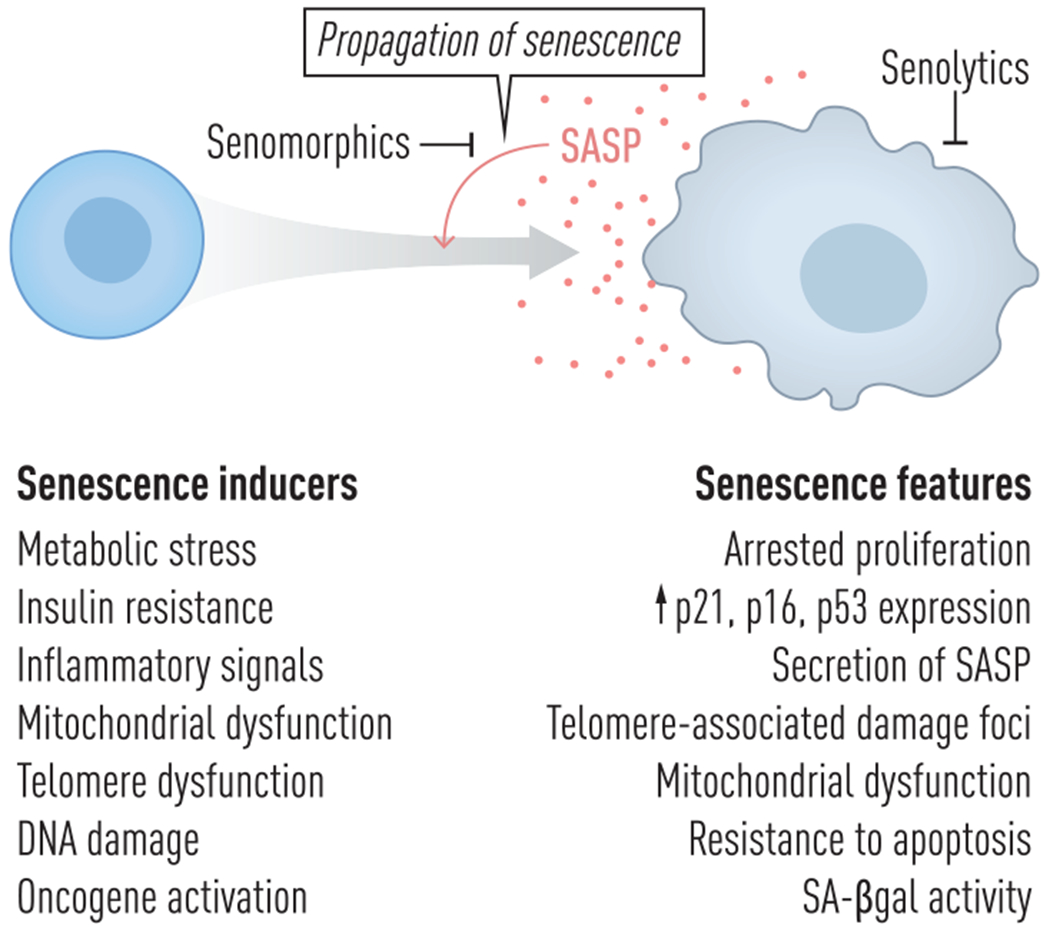
Inducers and features of senescent cells. Cells undergo senescence in response to a variety of stimuli, including metabolic stress, inflammatory signals, insulin resistance, telomere dysfunction, DNA damage, oncogene activation, and mitochondrial dysfunction. Typical features of most senescent cells are listed. In addition to being a defining feature of cellular senescence, the SASP, which is depicted by small circles here, is involved in signaling that leads to propagation of senescence in neighboring and distant cells. SASP, senescence-associated secretory phenotype.

As organisms age, cells can enter the state of senescence—a form of essentially irreversible growth arrest that typically occurs in response to cellular stressors such as repeated replication, DNA damage, metabolic or oxidative stress, inflammation, or telomeric dysfunction (Fig. 1) [1]. Senescence-triggering stimuli activate several key signaling pathways that lead to arrest of cell division. Among these are p16^INK4a^-, DNA damage response (DDR)-, and p53/ p21^CIP1/WAF1^-related pathways that can initiate and sustain the growth-arrested state of SCs. Although SCs no longer divide, they remain viable and metabolically active, secreting a range of bioactive molecules collectively known as the SASP. This can include inflammatory cytokines, chemokines, pro-fibrotic factors, proteases, growth factors, bioactive lipids, carbohydrates, reactive metabolites, and coding and non-coding nucleotides. These SASP factors can disrupt tissue homeostasis, induce senescence in neighboring and distant cells, and attract, activate, and anchor immune cells that further alter the tissue and whole-body environment [1–3]. Phenotypes of SCs are cell type- and tissue context-dependent. There are multiple markers for SCs of differing specificity and sensitivity. High protein levels of p53/p21^CIP1/WAF1^ and/or p16^INK4a^/Rb and DNA damage foci (particularly in telomeric regions—telomere-associated DNA damage foci [TAFs]), along with senescence-associated-*β*-galactosidase (SA-*β*gal) and increases in SASP factors, are the most widely used markers of SCs in solid tissue and sometimes liquid biopsies (blood, urine, cerebrospinal fluid, etc.). For instance, p16^INK4a^ expression in peripheral blood T cells and circulating SASP-associated proteins can reflect SC burden in animal models and humans [4–6]. Because none of these markers are fully sensitive and specific indicators of SCs on their own, composite scores may more accurately reflect SC abundance and target engagement in clinical trials of agents targeting SCs [6–10].

SCs can accumulate in multiple tissues in older individuals, as well as in individuals across the age range, with a number of disorders and diseases. In healthy younger individuals, the immune system efficiently clears SCs shortly after they appear, preventing SC persistence and accumulation [11]. Both formation of SCs and declines in immune surveillance can contribute to SC accumulation. The abundance of accumulated SCs can reach a “threshold” over which a feed-forward cycle of SASP-driven spread of senescence and consequent impairment of immune function is initiated, accelerating tissue degeneration with increases in multiple medical disorders within individuals (multimorbidity) and deteriorating health [12, 13]. Even a small number of SCs (<1% of total cells in the body) can exert widespread systemic effects. Additionally, in states of increased SC accumulation, such as in first-degree relatives of individuals with T2D, the percentage of SCs in subcutaneous AT is approximately or less than 10. In mice, the transplantation of a relatively low number of SCs (0.5–1 million) into young animals is sufficient to induce physical dysfunction and shorten lifespan because of early development of multiple diseases, highlighting the potent impact of the SASP on whole-organism physiology [5].

Senescence is not only a cell-autonomous phenomenon but can also propagate to neighboring and even distant cells, contributing to the spread of tissue dysfunction. One of the primary mechanisms underlying this propagation is the SASP, which can induce paracrine and endocrine senescence (Fig. 1) [14–18]. Another involves mitochondrial dysfunction and release of mitochondrial DNA (mtDNA) as part of the SASP. This activates the cGAS–STING pathway, promoting Type I interferon signaling and reinforcing senescence in the originating, neighboring, and distant cells [2, 19–21]. In some contexts, SCs may also transfer damaged mitochondria to neighboring cells by extracellular vesicles or tunneling nanotubes, inducing oxidative stress and DNA damage that can trigger senescence in recipient cells [22]. These findings highlight the capacity of senescence to propagate through both soluble inflammatory signals and intercellular organelle transfer, amplifying its impact across tissues and organ systems.

The immune system plays a vital role in clearing SCs to maintain tissue homeostasis and prevent age-related dysfunction. Natural killer (NK) cells recognize SCs by factors, such as NKG2D ligands, and eliminate SCs through cytotoxic granules containing perforin and granzymes, as occurs during liver fibrosis resolution [23]. Macrophages participate by phagocytosing SCs, a process aided by the SASP, which recruits immune cells to sites of senescence [24, 25]. However, SCs can evade immune-mediated clearance by upregulating inhibitory molecules such as HLA-E—which binds the NKG2A receptor on NK and CD8^+^ T cells to inhibit cytotoxic responses—and by expressing “don’t eat me,” “don’t find me,” and checkpoint signals such as CD47, GPNMB, and PD-L2, which block innate and adaptive immune-mediated SC removal [11, 26–28]. These immune evasion strategies can contribute to the age- and disease-related accumulation of SCs.

Mitochondrial dysfunction is a key component of the senescence phenotype. Senescence can be induced by telomere uncapping, which causes mitochondrial dysfunction [29, 30]. Reactive oxygen species is upstream of NF-*κ*B, which is an inducer of senescence [31, 32]. In addition, SCs are less able to tolerate uncoupling agents (such as FCCP) than non-SCs [33].

Nonproliferating cells can acquire a senescent-like state in response to stress or as a failed attempt to replicate. In these cells, senescence may be a protective mechanism to prevent persistence or replication of damaged DNA or to curb the harmful effects of chronic stress, but it may also represent a dysfunctional response to the inability to reenter the cell cycle. Endoreplication in nonproliferating cells caused by metabolic stress can occur in adipocytes and possibly neurons [34–37].

### Cellular senescence is increased in metabolic tissues in obesity and diabetes

Common human metabolic disorders are associated with increased cellular senescence in key insulin-regulated target cells, even after adjusting for the contributing effect of aging and BMI/obesity. These include diabetes, liver steatosis, and liver fibrosis (MASLD/MASH), which are common in both obesity and diabetes as well as cardiometabolic disorders [38]. The increased cellular senescence further promotes the clinical impact of the underlying disorders by enhancing inflammation, together with additional consequences of SASP factors. These include accentuated insulin resistance and hyperglycemia because pancreatic *β*-cells also become increasingly senescent with reduced ability to secrete insulin, and SASP factors impair insulin receptor signaling in metabolic cells. Large clinical studies in obese individuals have also documented that SC abundance in AT is positively associated with the degree of insulin resistance, dyslipidemia, and prevalence of T2D [39]. These results suggest that the potential to use therapies to target cellular senescence as a treatment for obesity and diabetes is logical, but the possibility that this will lead to a reduction in the number of pancreatic *β*-cells is a concern that needs to be addressed.

#### Human pancreatic β-cells

High rates of pancreatic *β*-cell replication occur during postnatal growth, which can lead to DNA damage and induction of cellular senescence. The increased abundance of human senescent pancreatic *β*-cells observed in diabetes can be promoted by hyperglycemia itself and by the associated hyperinsulinemia [40, 41]. Prolonged hyperinsulinemia due to insulin resistance or antidiabetic medication can induce senescence in human liver, adipose, and pancreatic *β*-cells. Increased cellular IGF1 can also promote senescence in pancreatic *β*-cells [42]. Insulin and IGF1 can activate Akt and its downstream signaling pathways that promote the development of cellular senescence [43–46]. Thus, cellular senescence may enable and fuel a vicious cycle of insulin resistance and hyperinsulinemia, both of which play important roles in the development of several metabolic diseases and their consequences.

An interesting question is whether physical exercise, which reduces insulin resistance and hyperinsulinemia, has positive effects on senescence in pancreatic *β*-cells and elsewhere. In a recent study, it was found that repeated exercise attenuated senescence in pancreatic *β*-cells, possibly because of increased glucagon levels, which increased AMP-activated protein kinase (AMPK) activity [47]. Furthermore, serum from exercise-trained human individuals contained factors that reduce the senescence of human pancreatic *β*-cells. We found that training reduces senescence in human skeletal muscle (SkM) cells and improves insulin sensitivity [48]. Together, these data support positive effects of some types of physical training on insulin resistance and glucose control, which, in turn, is promoted by reduced senescence in both pancreatic *β*-cells and peripheral metabolic cells.

#### Adipose tissue

White AT is one of the largest organs in the body. Adipose metabolism and secretion of signaling molecules are important regulators of lipid and carbohydrate metabolism as well as insulin sensitivity [49, 50]. Adipose cells are predisposed to undergo senescence with aging, obesity, and hyperinsulinemia [36, 49, 51]. This is further promoted in T2D. Adipose senescence may be due in part to hyperglycemia, but also to other factors such as endogenous hyperinsulinemia. Nevertheless, senescence is increased in T2D adipose cells independently from age and BMI [52]. It is not clear whether this is due to the adipose microenvironment or intrinsic/genetic effects, such that individuals with T2D are more prone to develop cellular senescence because of accelerated endogenous cellular aging processes.

Senescence has effects on undifferentiated and fully differentiated adipose cells. It prevents the normal differentiation of preadipocytes and increases the inflammation and secretion of SASP factors [53–55]. Senescence can also be induced in fully differentiated adipose cells, which do not undergo mitosis, in both obesity and T2D, in which the associated hyperinsulinemia plays an important role [35]. Indeed, hyperinsulinemia is a regulator of senescence in most if not all human metabolic cells, and this is mediated by the pro-mitogenic effect of insulin, like that of IGF1 [36, 41, 56].

Several clinical studies have shown that the extent of insulin resistance, hyperglycemia, and obesity in vivo correlates with increased levels of SCs in subcutaneous AT [35, 52, 53]. Due to its large size and ability to become senescent following adipose expansion, AT is considered to be a major contributor of the negative consequences of cellular senescence, even in metabolically healthy individuals [57, 51, 58, 59]. Interestingly, sodium–glucose cotransporter 2 inhibitor (SGLT2is) antidiabetic agents seem to have positive effects on adipose cellular senescence, and this may be one mode of action of these drugs [60].

#### Liver cells

Senescent hepatocytes alter liver homeostasis and microenvironment through SASP factors, which contribute to the development of metabolic dysfunction–associated steatotic liver disease (MASLD), fibrosis, cirrhosis and, thus, hepatocellular carcinoma [36, 61, 62]. Growing evidence suggests that SASP factors contribute to the development of liver steatosis, likely through mitochondrial dysfunction and impaired lipid metabolism. Indeed, the genetic removal of senescent hepatocytes improved hepatic steatosis in old, obese, and diabetic mice [63]. Senescent hepatocytes feature accumulation of damaged mitochondria and genomic instability and loss of proliferative and regenerative capacity. They also have telomere shortening and, like all SCs, dysregulation of the p53, p21, and p16 signaling pathways.

Increased steatosis is promoted by the dys-metabolic state, inducing cellular senescence. Again, insulin resistance and associated hyperinsulinemia play a significant role in the development of cellular senescence, which is also promoted by impaired lipid metabolism and steatosis. Animal experiments have indicated that senescence is promoted by increased lipid accumulation in liver cells and that senolytic therapy reduces both SCs and lipid accumulation [64]. It should also be emphasized that senescence not only develops in hepatocytes but also in hepatic stellate and Kupffer cells, further contributing to inflammation and fibrosis [65].

#### Skeletal muscle cells

SkM cells, including progenitor satellite cells, can become senescent, and this contributes to loss of SkM mass and strength with age [66]. SkM cells are also regulators of insulin-stimulated glucose uptake, indicating that increased cellular senescence contributes to impaired glucose tolerance with aging. Increased senescence in SkM cells also leads to reduced insulin signaling and action, as in adipose and many other cell types implicated in metabolic diseases [59].

Physical exercise appears to enhance insulin sensitivity, and recent studies have also indicated that exercise can reduce SC abundance in human SkM [48]. This is most evident in obese and older individuals, but also in younger individuals. Thus, SCs in SkM may be a target of physical exercise and certain antidiabetic medications such as glucagon-like peptide-1 (GLP-1) agonists and, to some extent, metformin [67, 68].

In summary, SCs are increased in pancreatic *β*-cells and key insulin-regulated metabolic organs with aging, obesity, and diabetes and contribute to inflammation and associated insulin resistance, hyperglycemia, and dyslipidemia, representing an opportunity for therapeutic development for these disorders.

### Therapeutic opportunities for targeting senescence for complications of diabetes

In addition to playing major roles in classically insulin-responsive tissues, cellular senescence has emerged as a contributor to the pathogenesis of diabetic complications across multiple other organ systems. In the context of diabetes, chronic hyperglycemia, oxidative stress, and metabolic dysregulation drive the accumulation of SCs, which in turn secrete pro-inflammatory and tissue-degrading factors that disrupt organ function. Cellular senescence contributes to the progression of diabetic complications in the kidney, retina, heart, skin, and other organs, highlighting its role in inflammation, fibrosis, and impaired regeneration.

#### Diabetic nephropathy

In diabetic nephropathy, high glucose levels induce premature cellular senescence in renal tubular epithelial cells, leading to renal interstitial fibrosis and impaired kidney function [69]. Markers of cellular senescence, such as p21 and p16, are elevated in diabetic kidney tissues, indicating accelerated cellular aging [70, 71]. This senescent state contributes to the deterioration of kidney structure and function, exacerbating the progression of diabetic kidney disease [72].

#### Retina

In the retina, hyperglycemia-induced senescence of endothelial cells contributes to the pathogenesis of diabetic retinopathy [73]. Senescent endothelial cells exhibit a pro-inflammatory phenotype, disrupting vascular integrity and promoting pathological angiogenesis [69]. This vascular dysfunction can contribute to retinal damage and vision loss, commonly observed in diabetic retinopathy.

#### Heart

Cellular senescence plays a significant role in age-related cardiac dysfunction, as various heart cell types—including cardiomyocytes, endothelial cells, fibroblasts, and progenitor cells—can enter a senescent state in response to stress and acquire an SASP that disrupts tissue homeostasis and promotes pathological remodeling [74]. In diabetic cardiomyopathy, SCs exacerbate myocardial inflammation and fibrosis. Hyperglycemia-induced oxidative stress and mitochondrial dysfunction drive cardiomyocyte senescence, leading to structural and functional changes in the heart. Furthermore, senescent endothelial cells impair vascular function by reducing nitric oxide availability, which promotes atherosclerosis and compromises cardiac perfusion [75, 76].

#### Duodenum

Resurfacing of the duodenal mucosa has been shown in preliminary studies to improve metabolic phenotypes in diabetic individuals [77]. Whether this effect is related to ablation of SCs is an interesting possibility that is being investigated.

#### Skin

Individuals with diabetes or metabolic syndrome often experience skin complications, including delayed wound healing, chronic ulcers, increased susceptibility to infections, and skin thinning [78]. These issues have been attributed to factors such as chronic hyperglycemia, insulin resistance, and systemic inflammation, which impair skin integrity and function [79]. Cellular senescence contributes to skin dysfunction by promoting a pro-inflammatory environment and impairing tissue regeneration. In diabetic conditions, elevated glucose levels and oxidative stress induce cellular senescence in skin cells, including fibroblasts and keratinocytes. SASP factors secreted by SCs can disrupt normal skin architecture and healing processes [80]. For example, the accumulation of SCs in diabetic skin impairs wound healing by inhibiting cell proliferation and promoting chronic inflammation. This leads to persistent, non-healing wounds that are prone to infection and may result in complications such as ulcers or amputations [81].

#### Bladder

Diabetic bladder dysfunction (DBD) is a prevalent complication of diabetes, affecting up to 50% of patients [82]. DBD encompasses a spectrum of lower urinary tract symptoms, including urgency, frequency, incontinence, and incomplete bladder emptying. Similarly, metabolic syndrome has been linked to an increased risk of overactive bladder symptoms, suggesting shared pathophysiological mechanisms between these metabolic conditions and bladder dysfunction [83]. Cellular senescence is hypothesized to contribute to bladder dysfunction in metabolic diseases. In the context of diabetes, studies have identified increased cellular senescence markers in the urothelial layer of the bladder. These SCs can disrupt normal bladder function by promoting inflammation, oxidative stress, and tissue remodeling. In a preclinical model of aging, umbrella cells, luminal barrier uroepithelial cells in the bladder, exhibit senescence features and are increased after high-fat feeding [84]. Interestingly, initial studies indicate that these cells are not susceptible to the senolytic combination of dasatinib and quercetin; however, additional work is needed to determine whether these cells have unique features that may render them susceptible to alternative senotherapeutic strategies.

Targeting cellular senescence presents a promising therapeutic avenue for mitigating diabetic complications. Interventions aiming to eliminate SCs or modulate their secretory profiles are under investigation to improve the function of end organs. These strategies hold potential for preserving organ function and enhancing quality of life in individuals with diabetes.

### Effect of antidiabetic agents on cellular senescence

Mounting evidence indicates that hyperinsulinemia plays a causal role in establishing a vicious cycle that promotes cellular senescence, which in turn exacerbates insulin resistance through SASP-mediated inflammation and tissue dysfunction [36, 85, 86]. This interplay positions hyperinsulinemia as both a driver and consequence of cellular senescence, providing a mechanistic rationale for antidiabetic therapies that lower insulin levels as potential senotherapeutic agents.

Preclinical studies indicate that reducing circulating insulin levels—such as through insulin gene dosage modulation—enhances insulin sensitivity and extends lifespan in mice [87]. In obese mammals, preventing chronic hyperinsulinemia or lowering basal insulin levels improves insulin resistance, hepatic steatosis, and inflammation while extending lifespan [88]. In humans, insulin hypersecretion is an early predictor of metabolic decline: Individuals with normal glucose tolerance but high insulin secretion have progressive deterioration in insulin sensitivity and adverse metabolic trajectories over time [89]. Longitudinal data from the Baltimore Aging Study further support this, showing that lower plasma insulin levels are associated with increased survival [90].

Across metabolic tissues such as AT and liver, hyperinsulinemia strongly correlates with both insulin resistance and cellular senescence in obesity, T2D, and MASLD [35, 41, 53, 91]. Experimental models corroborate a causal role of insulin in cellular senescence induction in these contexts [35, 41, 53, 91]. Reciprocally, SCs directly drive insulin resistance, as shown by the metabolic improvements observed following senolytic treatments in preclinical models of aging and metabolic disease [13, 92].

Several promising therapeutic strategies have been identified that lower insulin levels, attenuate cellular senescence, and improve insulin sensitivity (Table 1). Among these are palmitic acid esters of hydroxy stearic acids (PAHSAs)—a novel class of endogenous bioactive lipids with insulin-sensitizing and anti-inflammatory properties [93, 94]. PAHSAs have been shown to exert anti-senescent effects in pancreatic islets from both humans and mice, supporting their potential as modulators of cellular senescence in metabolic tissues [95]. Mechanistically, PAHSAs counteract cellular senescence by reducing p53 activity and DNA damage, preventing cells from prematurely entering a senescent state. Additionally, PAHSAs stimulate autophagy, facilitating the clearance of damaged proteins and organelles that would otherwise contribute to senescence. Importantly, PAHSAs also suppress the SASP, thereby mitigating the inflammatory feedback loop that drives propagation of cellular senescence across tissues [95]. In preclinical models, PAHSA treatment improves glucose tolerance, enhances insulin signaling, and lowers inflammatory markers, suggesting that these lipids may help prevent or reverse pancreatic *β*-cell dysfunction in diabetes [96]. The beneficial effects of PAHSAs extend beyond direct *β*-cell protection, as they also modulate immune responses, reducing systemic inflammation and promoting metabolic homeostasis [93, 94, 96]. Evidence from human studies further supports the role of PAHSAs in metabolic regulation, as circulating PAHSA levels correlate positively with insulin sensitivity [93]. Notably, exercise has been shown to increase PAHSA levels in both serum and AT of elderly women, suggesting that PAHSAs may act as mediators of the metabolic benefits associated with physical activity [97, 98]. Given their favorable safety profile and ability to engage multiple pathways implicated in aging and metabolic dysfunction, PAHSAs represent a promising therapeutic strategy for mitigating senescence-related metabolic diseases. However, further research is required to determine the most effective methods for increasing PAHSA levels, either through dietary supplementation, pharmacological intervention, or lifestyle modifications.

Although PAHSAs remain at an experimental stage, their insulin-sensitizing and anti-senescence effects provide compelling proof-of-concept that targeting hyperinsulinemia and its downstream consequences can modulate cellular senescence. This rationale is already exemplified by FDA-approved antidiabetic agents—including SGLT2is, GLP-1 receptor agonists (GLP-1RAs), and dipeptidyl peptidase-4 inhibitors (DPP-4is), and possibly metformin—which engage the same core pathways and therefore hold immediate translational and clinical promise. Beyond glycemic control, these drugs act directly on the cellular mechanisms that link aging to metabolic disease: By reducing SC burden across key tissues, they have the potential to disrupt the vicious cycle of hyperinsulinemia, insulin resistance, and tissue dysfunction, thereby delivering the dual benefit of improved metabolic regulation and mitigation of age-related cellular damage. SGLT2is have revolutionized diabetes management, demonstrating profound benefits beyond glucose control, including protection against cardiovascular and kidney diseases, MASLD regression, and mortality reduction [99–101]. Recent findings suggest that SGLT2is may also exert senotherapeutic effects, making them a promising class of drugs in metabolic diseases that intersect with fundamental aging pathways [60]. Unlike traditional glucose-lowering agents, SGLT2is promote a fasting-mimicking state, reducing insulin levels while enhancing lipid oxidation and ketogenesis [102]. By lowering chronic hyperinsulinemia, SGLT2is may mitigate insulin-induced cellular senescence, thereby improving metabolic flexibility and tissue function. Emerging evidence suggests that SGLT2is facilitate immune-mediated clearance of SCs, particularly in metabolically active tissues such as the liver and AT. Short-term treatment with canagliflozin, a widely used SGLT2i, has been shown to reduce SC burden across multiple tissues [103]. Mechanistic studies indicate that canagliflozin enhances autophagy, activates AMPK, and suppresses programmed cell death ligand 1 (PD-L1) expression on SCs, thereby restoring immune surveillance and SC elimination by NK and CD8^+^ T cells [60]. Notably, those SCs with the highest PD-L1 expression also exhibit increased SASP activity, highlighting a particularly deleterious subset that may be preferentially targeted by SGLT2is [60]. Future studies should explore the long-term effects of SGLT2is on senescence-related metabolic dysfunction and evaluate their potential in combination therapies for age-related diseases.

GLP-1RAs and DPP-4is have gained prominence in diabetes therapy due to their ability to enhance insulin secretion, promote weight loss, and reduce cardiovascular risk [104]. Beyond their glucose-lowering effects, these drugs exhibit anti-senescence and anti-inflammatory properties, making them attractive candidates for targeting aging-related metabolic dysfunction [105, 106]. GLP-1RAs function by activating AMPK and nuclear erythroid 2–related factor 2 (NRF2), two key regulators of cellular stress responses that inhibit senescence. Preclinical data show that exendin-4, a prototypical GLP-1RA, reduces senescence in pancreatic *β*-cells and reverses age-related transcriptomic changes in AT, liver, skeletal muscle, and circulating leukocytes [47]. DPP-4is, which prolong the bioavailability of endogenous GLP-1, also appear to exert direct anti-senescent effects. Studies in human cell models show that DPP-4is attenuate premature senescence induced by metabolic or oxidative stress [107–109]. Consistent with this, clinical studies have indicated that long-term use of GLP-1RAs and DPP-4is is associated with reduced systemic inflammation and a lower risk of metabolic decline, contributing to many diabetes comorbidities, such as MASLD.

Metformin is an antidiabetic that appears to affect multiple age-related processes. The proposed but not yet active TAME trial will investigate its potential to extend healthspan and delay age-related diseases beyond glycemic control [110]. Mechanistically, metformin activates AMPK, enhancing mitochondrial function and reducing oxidative stress—key drivers of cellular senescence. Additionally, metformin suppresses NF-*κ*B signaling, mitigating the SASP and chronic inflammation, central to senescence-associated metabolic dysfunction [110, 111]. By improving insulin sensitivity, metformin may break the cycle of hyperinsulinemia-driven senescence. However, its senotherapeutic efficacy likely depends on dose and tissue specificity, warranting much further investigation.

Together, these insights reframe antidiabetic agents not as only metabolic regulators but also emerging senotherapeutics. By linking experimental discoveries such as PAHSAs with established therapies already in clinical use, this paradigm highlights the translational opportunity of repurposing antidiabetic drugs to target senescence. Such an approach paves the way for a new era in metabolic disease management, where integrating senescence-targeting strategies with current standards of care may simultaneously improve metabolic control, extend healthspan, and delay the progression of age-related disorders.

### Lifestyle interventions mimicking antidiabetic therapies

The core mechanisms harnessed by antidiabetic agents—lowering hyperinsulinemia, enhancing insulin sensitivity, and mitigating cellular senescence—are also engaged by lifestyle interventions. This positions lifestyle approaches as their physiological counterpart and a natural basis for synergy, while underscoring their translational relevance as a cornerstone of metabolic disease prevention and treatment.

Exercise is an effective natural intervention for counteracting cellular senescence and metabolic dysfunction [48]. Regular physical activity exerts systemic anti-senescence effects by lowering plasma insulin levels, alleviating insulin resistance, enhancing mitochondrial function, and reducing inflammation across multiple tissues. Exercise engages endogenous protective mechanisms that not only reduce the burden of SCs but also prevent their accumulation [47, 112]. One of the primary ways in which exercise modulates senescence is by activating AMPK, which inhibits pro-senescence pathways while promoting mitochondrial biogenesis and autophagy. Exercise also triggers the activation of NRF2, enhancing antioxidant defenses [47]. By improving glucose uptake and reducing insulin demand, exercise mitigates hyperinsulinemia, thereby preventing insulin-induced cellular senescence. Preclinical models suggest exercise decreases pancreatic islet senescence by increasing GLP-1 levels, activating AMPK, and engaging NRF2 [47]. These beneficial effects were also observed in human pancreatic islets. Notably, serum from exercise-trained diabetic individuals reduces SC marker expression in human pancreatic *β*-cells, suggesting that exercise induces circulating factors that target SCs [47]. These findings are supported by clinical studies demonstrating SC reductions in blood and SkM following regular physical activity [48, 112–114]. Although its metabolic effects are well established, future research should focus on optimizing exercise regimens for maximizing senotherapeutic effects and exploring the potential synergy between exercise and senolytic therapies.

Caloric restriction (CR) shares key benefits with exercise, including delaying metabolic disorders such as obesity and T2D [115]. Its effects are largely mediated by reduced insulin levels and improved insulin sensitivity, but underlying mechanisms remain unclear [116]. Notably, CR lowers SC markers across multiple tissues, suggesting that the reduction of SCs is a likely contributor [117]. Like physical exercise, a key mechanism through which CR counteracts senescence is AMPK activation, which enhances autophagy and limits SC accumulation by inhibiting pro-survival pathways. CR also reduces pro-inflammatory cytokine production, curbing SASP-induced senescence and preserving tissue integrity and metabolic function [117]. Additionally, CR may enhance immune-mediated clearance of SCs by reversing seno-energy [28, 118]. Moreover, CR lowers circulating insulin, which may contribute to the CR-induced mitigation of cellular senescence and insulin resistance. In rodent models, lifelong CR reduces SC burden in AT, liver, and pancreas, improving metabolic outcomes [119–121]. Human studies further support these findings: 12- or 24-month interventions with moderate CR significantly reduced senescence biomarkers in both plasma and AT in young-to-middle-aged adults, with improvements in insulin sensitivity and overall metabolic health [122]. Despite its well-established benefits, long-term adherence to CR is challenging in modern obesogenic environments. This has fueled interest in CR mimetics—compounds that replicate its metabolic and anti-senescence effects without the need for sustained caloric reduction. Notably, some of the most promising CR mimetics are already used as antidiabetic therapies, such as SGLT2is and metformin, directly linking lifestyle interventions to drug-based strategies and underlining their convergence on shared pathways. Together, these findings highlight antidiabetic agents as a promising new class of senotherapeutics, with the potential to improve metabolic control while reducing SC burden. Lifestyle interventions engage overlapping mechanisms and can act synergistically with pharmacological strategies. This perspective underscores the translational relevance of repositioning antidiabetic therapies as senescence-targeting interventions. Table 1 offers a concise framework through which both antidiabetic agents and lifestyle approaches can be viewed as senotherapeutic strategies in metabolic disease.

### Senolytics and senomorphics

Senolytic agents represent a promising class of interventions aimed at selectively eliminating SCs. Initial discoveries identified compounds such as dasatinib and quercetin, which target anti-apoptotic pathways that SCs rely on for survival. These pathways—termed SC anti-apoptotic pathways (SCAPs)—provide a survival advantage to SCs, allowing them to resist apoptosis despite their detrimental effects on neighboring cells and tissue function. By transiently inhibiting SCAPs, senolytics can induce apoptosis in those SCs that release pro-apoptotic factors without affecting normal cells [123–128], offering a targeted approach to mitigating the multiple pathologies related to cellular senescence across the lifespan [12, 74, 129–131]. Senomorphics are agents that inhibit the release of some or multiple SASP factors by SCs—such as metformin, agents related to rapamycin, JAK/STAT inhibitors, and agents targeting histone H3-specific demethylases, such as the KDM4 modulator ML324 [132–135]. Some agents—such as zoledronate, which is used for treating bone disorders—are both senolytic and senomorphic [132].

Preclinical studies have indicated that senolytic treatment might be beneficial across a range of conditions. In animals and human cell culture, organoid, and tissue explant models, senolytics have been shown to delay, prevent, alleviate, or treat diabetes and obesity and their complications: cardiac, vascular, renal, dermatological, gastrointestinal, and visual dysfunction; osteoporosis, MASH and MASLD; cognitive disorders; cancers; and morbidity related to infectious, rheumatologic, and pulmonary disorders, among many others [74, 128–131, 136–139]. These benefits are attributed to the reduction of SC burden and the consequent decrease in pro-inflammatory, tissue-damaging, pro-fibrotic SASP factors. Notably, the effects of senolytics occur with intermittent dosing, as SCs take time to reaccumulate, allowing for a “hit-and-run” therapeutic strategy [12].

The translation of senolytic therapies into clinical settings is underway, with early phase trials exploring their safety and efficacy in humans. For example, an open-label trial involving a 3-week course of dasatinib and quercetin demonstrated improvements in physical function (6-min walk distance, gait speed, and chair-stand time) among patients with idiopathic pulmonary fibrosis [140]. Another trial targeting diabetic kidney disease indicated reductions in SC markers and inflammatory cytokines in AT after a short course of treatment with dasatinib and quercetin [10]. These studies had excellent retention rates reflecting good patient tolerance of therapy, with few adverse events. Clinical trials of senolytics for Alzheimer’s disease and age-related osteoporosis suggest early promise [141–144]. These findings point to senolytics having broad therapeutic potential across a number of human disorders and diseases. If so, this would support the geroscience hypothesis that targeting fundamental aging processes can simultaneously impact multiple conditions.

### Novel hypotheses for targeting senescent cells: senosensitizers and chemosensitizers

**Fig. 2:**
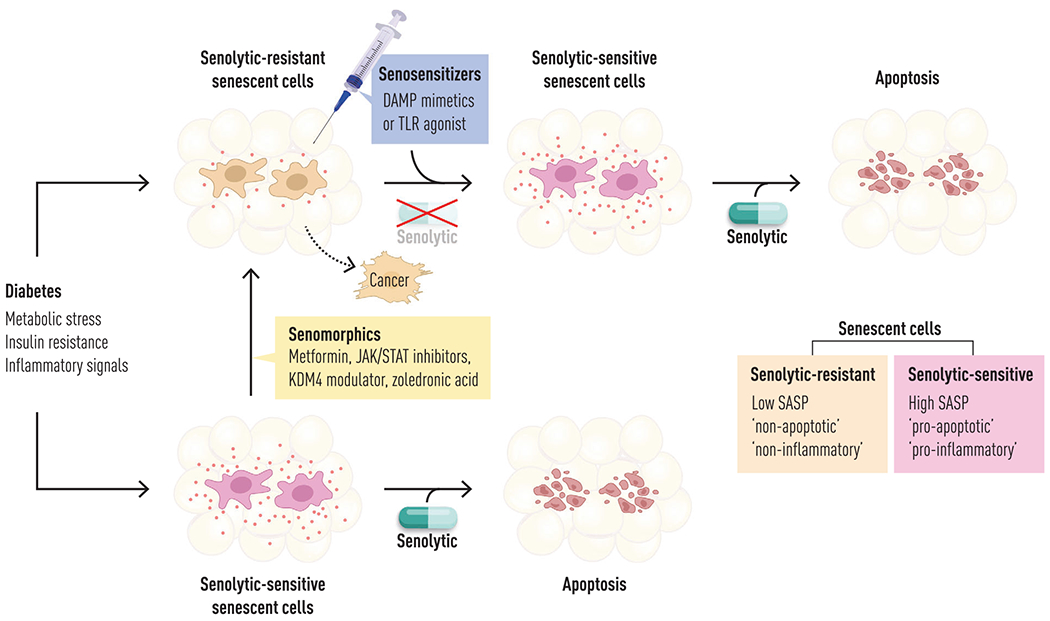
Senolytic-sensitive SCs, senolytic-resistant SCs, and senosensitizers. There are two major types of SCs: (1) pro-apoptotic, pro-inflammatory SCs, which are sensitive to senolytics; and (2) non-apoptotic, non-inflammatory SCs, which are resistant to senolytics. Senosensitizers and related chemosensitizers, including DAMP mimetics or TLR agonists, are emerging therapeutic agents capable of converting these senolytic-resistant SCs—potentially harboring oncogenic mutations—into senolytic-sensitive cells, thereby enabling their targeted elimination by senolytics. DAMP, damage-associated molecular profile factor; SC, senescent cell; TLR, toll-like receptor.

**Fig. 3:**
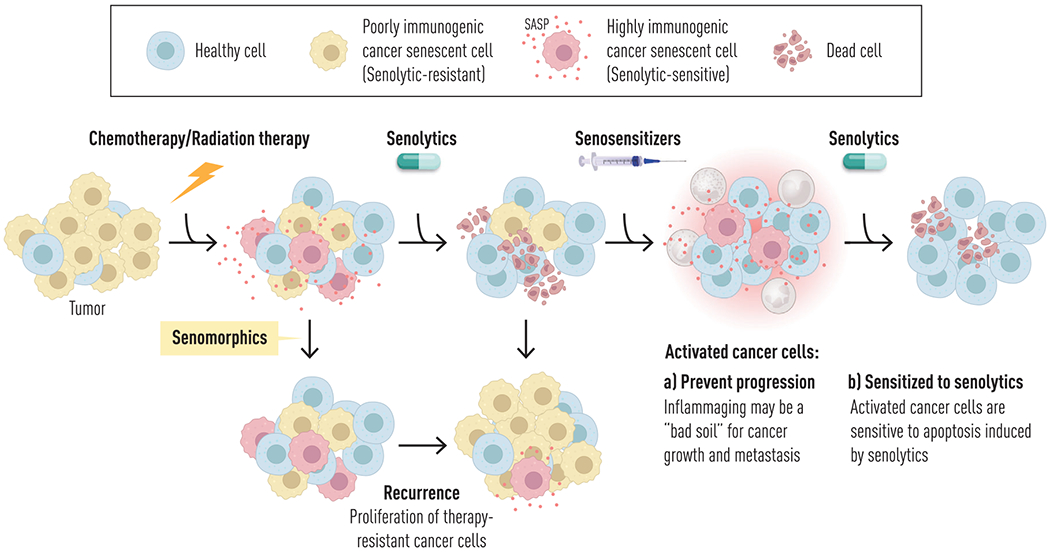
“1-2-3-4 Punch” strategy to target chemotherapy-resistant cancer cells and control cancer progression. The “1-2-3-4 punch” approach is a conceptual therapeutic strategy proposed to enhance cancer and potentially metabolic disease control: (1) Chemotherapy and radiation therapy induce senescence in cancer cells (therapy-induced senescence). (2) Senolytics, many of which also have chemotherapeutic activity, selectively eliminate pro-apoptotic, senescent cancer cells. (3) Senosensitizers convert senolytic-resistant, non-apoptotic senescent cancer cells into senolytic-sensitive cells. (4) A second round of senolytic treatment removes these previously resistant cells. Senosensitizers not only enhance the efficacy of senolytics to remove cancers (remove “seeds”) but may also function as chemosensitizers, thereby augmenting the effects of senolytics after chemotherapy and radiation. In addition, by inducing a controlled pro-inflammatory state, senosensitizers may create a microenvironment (“bad soil”) that is hostile to cancer growth and metastasis, potentially preventing relapse.

Overall, 30%–70% of SCs are resistant to the first generation of senolytic agents that transiently disable SCAPs [123]. These senolytic-resistant SCs still have an SASP, but it tends not to be pro-apoptotic and pro-inflammatory. Senosensitizers and related chemosensitizers are novel agents that convert these non-apoptotic, non-inflammatory but potentially cancerous mutation-harboring “ticking time bomb” SCs as well as cancer cells, into SCs or cancer cells that are more susceptible to senolytics, chemotherapy, and removal by the immune system (Fig. 2; publications submitted). Factors that appear to cause senolytic-resistant cells to convert into being pro-apoptotic (and hence senolytic- and chemotherapy-sensitive) and pro-inflammatory (and hence susceptible to elimination by the immune system) include damage-associated molecular profile factors (DAMPs), pathogen-associated molecular patterns (PAMPs), and inflammatory cytokines. We reported that PAMPs and DAMPs—such as lipopolysaccharide (LPS; [145, 146]) and cell-free nucleotides (which act through toll-like receptors [TLRs; [147]] and other TLR agonists)—can convert non-inflammatory SCs into pro-inflammatory SCs with a pro-apoptotic SASP in less than an hour. Indeed, levels of some pro-apoptotic SASP factors increase by three orders of magnitude in human SCs following exposure to such senosensitizers, making them susceptible to senolytics (publications submitted). In keeping with our published findings, it appears cancer-harboring SCs with a non-inflammatory SASP and that express immune-evading signals—such as checkpoint inhibitors—can be converted into senolytic- and immune system-susceptible SCs and cancer cells by inflammation-mimicking senosensitizers/chemosensitizers (publication submitted). Moreover, in keeping with this, it appears that several types of cancers, once they occur, are more rapidly progressive (higher rates of growth and metastatic spread) in younger individuals than in older individuals with chronic low-grade inflammation. An example is triple-negative breast cancer, in which younger patients seem to have more aggressive tumors and increased growth of metastases in the liver and brain [148]. Furthermore, consistent with inflammaging conferring some resistance to cancer progression, very old individuals with multimorbidity whose immediate cause of death is from diseases other than cancers (e.g., falls, myocardial infarction) sometimes are found to have multiple cancers at autopsy that were not ostensibly symptomatic during life, with cancer incidence and mortality decreasing at very old age (with exceptions) [149]. Moreover, frailty in men is associated with decreased cancer incidence [150]. Hence, it is tempting to speculate that treatment with senosensitizers (e.g., DAMP mimetics or TLR agonists) and related chemosensitizers to heighten the inflammatory state of SCs or cancer cells, followed by senolytics/chemotherapy and/or checkpoint inhibitors, may have a role in delaying or slowing complications of SC accumulation and cancer progression (Fig. 3). Data are in submission supporting cycles of this “1-2-3-4” punch approach to control cancer progression and target chemotherapy-resistant cancer cells. Perhaps these findings and interventions may extend to targeting links between SC accumulation, cancers, and infectious complications in the context of obesity and metabolic diseases, a hypothesis being tested currently.

### Challenges and unanswered questions

Challenges remain in the clinical application of senotherapeutics for metabolic disorders and their complications. Identifying biomarkers to quantify SC abundance and treatment response is critical for patient selection and monitoring. Additionally, understanding the heterogeneity of SCs across different tissues and conditions is essential to optimizing therapeutic strategies and minimizing potential adverse effects. Ongoing research aims to address these challenges, refine the choice, doses, timing of administration, and sequencing of senolytic, senosensitizing, and immune checkpoint inhibiting compounds and combinations of senotherapeutics with lifestyle and disease-specific interventions. It also aims to establish standardized protocols to harness the full potential of senotherapeutics in delaying, preventing, alleviating, and treating senescence-linked metabolic diseases and their complications. Among many issues remaining to be investigated more fully are (1) heterogeneity of cellular senescence; (2) outcome criteria of senotherapeutic clinical trials for metabolic disorders, diseases, and their complications; (3) pharmacokinetic/dynamic issues; (4) imaging of SCs in vivo; (5) safety/tolerability of senotherapeutics; (6) effects of senotherapeutics on other fundamental aging processes; and (7) unrealistic and over-enthusiastic public perceptions and statements by investigators or companies with conflicts of interest that characterize senotherapeutics as “anti-aging” compounds that can “reverse” aging. In addition to promoting unrealistic expectations for interventions that remain under investigation and validation, these statements ignore the risk of tumorigenesis that could be created by preventing or reversing cellular senescence.

## Conclusions

Medicine is entering an era that involves clinical translation of basic to clinical studies of gerotherapeutics that may have a profound impact on multiple disorders and diseases across the lifespan. Among the most promising frontiers is the development of therapies that target persisting SCs and their harmful downstream effects. Early studies suggest that clearing or modulating the effects of SCs could transform treatment for diabetes, obesity, and other metabolic disorders, addressing not only the diseases themselves but also their wide-ranging complications. Beyond improving metabolic health, reducing SC burden may yield broader benefits, including lowering the risk of cancers and other serious comorbidities that disproportionately affect individuals with metabolic dysfunction.

## Figures and Tables

**Table 1.** Antidiabetic agents and lifestyle interventions as senotherapeutic strategies in metabolic disease.

Category	Intervention	Mechanism of action on senescence	Clinical/Metabolic effects	Evidence in humans	Translational significance
**Experimental molecules**	**PAHSAs**	↓p53/DNA damage; ↓SASP; ↑Autophagy; immune modulation	Preserve *β*-cell function; improve insulin signaling; reduce inflammation	Circulating PAHSA levels correlate with insulin sensitivity and are increased by exercise	Potential biomarker and therapeutic candidate
**Antidiabetic agents**	**SGLT2is**	↓Hyperinsulinemia; ↑Lipid oxidation; ↑Autophagy; restore immune clearance; mimic fasting	Reduce SC burden in liver and adipose tissue; improve metabolic flexibility; protect the kidney and heart	Clinical trials: ↓cardiovascular and renal risk, ↓mortality; MASLD regression; anti-senescence effects	First widely used antidiabetics with senotherapeutic activity
	**GLP-1RAs**	↑AMPK/NRF2; ↓Inflammation; protect *β*-cells	Reverse age-related changes in *β*-cells, adipose tissue, liver, skeletal muscle; improve insulin sensitivity	Clinical use is associated with reduced systemic inflammation and cardiovascular protection	Dual benefits by glycemic control while modulating aging- and senescence-related pathways
	**DPP-4is**	Prolong GLP-1 action; ↑AMPK/SIRT1/NRF2	Attenuate premature senescence in endothelial cells and *β*-cells	Clinical use is associated with reduced metabolic decline and systemic inflammation	Complementary to GLP-1RAs; growing mechanistic evidence
	**Metformin**	↑AMPK; ↑mitochondrial function; ↓NF-*κ*B/SASP	Improve insulin sensitivity; reduce oxidative stress and chronic inflammation	Epidemiological data: ↓Risk of multiple age-related disorders; TAME trial planned	Paradigmatic CR mimetic; candidate gerotherapeutic
**Lifestyle interventions**	**Exercise**	↓Insulin demand; ↑AMPK/NRF2; ↑Autophagy; ↑Circulating anti-senescence factor	Reduce SC burden in *β*-cells and skeletal muscle; improve insulin sensitivity and mitochondrial health	Clinical studies: ↓Senescence biomarkers in blood and muscle	Central component of diabetes management; physiological counterpart to drugs
	**CR**	↓Insulin; ↑Autophagy; ↓SASP; ↑Immune clearance of SCs	Lower SC burden in adipose tissue, liver, pancreas; improve systemic metabolism	Human trials: ↓Senescence biomarkers after 12–24 months of CR	Basis for CR mimetics; mechanistic template for pharmacological agents

*Note:* Experimental molecules, FDA-approved antidiabetic agents, and lifestyle interventions that target cellular senescence and its role in metabolic disease.

Abbreviations: AMPK: AMP-activated protein kinase; CR: caloric restriction; DPP-4is: dipeptidyl peptidase-4 inhibitors; GLP-1RAs: glucagon-like peptide-1 receptor agonists; MASLD: metabolic dysfunction-associated steatotic liver disease; NF-*κ*B: nuclear factor kappa-light-chain-enhancer of activated B cells; NRF2: nuclear factor erythroid 2–related factor 2; PAHSAs, palmitic acid esters of hydroxy stearic acids; SASP: senescence-associated secretory phenotype; SCs: senescent cells; SGLT2is, sodium–glucose cotransporter 2 inhibitors; TAME trial: targeting aging with metformin.

## Abbreviations:

AMPK: AMP-activated protein kinase
AT: adipose tissue
CR: caloric restriction
DBD: diabetic bladder dysfunction
DPP-4is: dipeptidyl peptidase-4 inhibitors
GLP-1: glucagon-like peptide-1
GLP-1RAs: GLP-1 receptor agonists
MASH: metabolic dysfunction–associated steatohepatitis
MASLD: metabolic dysfunction–associated steatotic liver disease
mtDNA: mitochondrial DNA
NF-*κ*B: nuclear factor kappa-B
NK: natural killer cells
NRF2: nuclear erythroid 2–related factor 2
PAHSA: spalmitic acid esters of hydroxy stearic acids
PD-L1: programmed cell death ligand 1
SASP: senescence-associated secretory phenotype
SGLT2is: sodium–glucose cotransporter 2 inhibitors
SCAPs: senescent cell anti-apoptotic pathways
SkM: human skeletal muscle
T2D: Type 2 diabetes
